# Altered Esophageal Histamine Receptor Expression in Eosinophilic Esophagitis (EoE): Implications on Disease Pathogenesis

**DOI:** 10.1371/journal.pone.0114831

**Published:** 2015-02-27

**Authors:** Jamie Merves, Prasanna Modayur Chandramouleeswaran, Alain J. Benitez, Amanda B. Muir, Anna J. Lee, Diana M. Lim, Kara Dods, Isha Mehta, Eduardo D. Ruchelli, Hiroshi Nakagawa, Jonathan M. Spergel, Mei-Lun Wang

**Affiliations:** 1 Division of Gastroenterology, Hepatology, and Nutrition, The Children’s Hospital of Philadelphia, Philadelphia, Pennsylvania, United States of America; 2 Division of Allergy and Immunology, The Children’s Hospital of Philadelphia, Philadelphia, Pennsylvania, United States of America; 3 Division of Pathology, The Children’s Hospital of Philadelphia, Philadelphia, Pennsylvania, United States of America; 4 Department of Pediatrics, Perelman School of Medicine at the University of Pennsylvania, Philadelphia, Pennsylvania, United States of America; 5 Department of Gastroenterology, Hepatology and Nutrition, Perelman School of Medicine at the University of Pennsylvania, Philadelphia, Pennsylvania, United States of America; Vanderbilt University, UNITED STATES

## Abstract

Eosinophilic Esophagitis (EoE) is a chronic allergic disorder, whose pathobiology is incompletely understood. Histamine-producing cells including mast cells and basophils have been implicated in EoE. However, very little is currently known about the role of histamine and histamine receptor (HR) expression and signaling in the esophageal epithelium. Herein, we characterized HR (H1R, H2R, H3R, and H4R) expression in human esophageal biopsies and investigate the role of histamine signaling in inducible cytokine expression in human esophageal epithelial cells *in vitro*. HR expression was quantified in esophageal biopsies from non-EoE control (N = 23), inactive EoE (<15 eos/hpf, N = 26) and active EoE (>15 eos/hpf, N = 22) subjects using qRT-PCR and immunofluorescent localization. HR expression and histamine-mediated cytokine secretion were evaluated in human primary and telomerase-immortalized esophageal epithelial cells. H1R, H2R, and H4R expression were increased in active EoE biopsies compared to inactive EoE and controls. H2R was the most abundantly expressed receptor, and H3R expression was negligible in all 3 cohorts. Infiltrating eosinophils expressed H1R, H2R, and H4R, which contributed to the observed increase in HR in active subjects. H1R and H2R, but not H3R or H4R, were constitutively expressed by primary and immortalized cells, and epithelial histamine stimulation induced GM-CSF, TNFα, and IL-8, but not TSLP or eotaxin-3 secretion. Epithelial priming with the TLR3 ligand poly (I:C) induced H1R and H2R expression, and enhanced histamine-induced GM-CSF, TNFα, and IL-8 secretion. These effects were primarily suppressed by H1R antagonists, but unaffected by H2R antagonism. Histamine directly activates esophageal epithelial cytokine secretion *in vitro* in an H1R dependent fashion. However, H1R, H2R and H4R are induced in active inflammation in EoE *in vivo*. While systemic antihistamine (anti-H1R) therapy may not induce clinical remission in EoE, our study suggests that further study of histamine receptor signaling in EoE is warranted and that targeting of additional histamine receptors may lead to novel treatment strategies for this important disease.

## Introduction

Eosinophilic esophagitis (EoE) is a chronic food antigen-mediated disease characterized by eosinophil infiltration into the esophageal epithelium [1]. EoE is evolving as one of the most common causes of chronic esophagitis in children and adults, with recent incidence estimates of 56.7 per 100,000 and up to 50.5 per 100,000 in children [2]. Current disease monitoring is limited to endoscopic surveillance and treatment options include dietary restrictions and long term swallowed corticosteroids. Thus, further understanding of this emerging disease is essential to develop less invasive monitoring strategies and additional treatment options.

The pathophysiology of EoE is incompletely understood, though both genetic predisposition and complex interactions between many inflammatory cells and pathways [3,4] are likely to be involved. Though the diagnostic hallmark of EoE is the infiltration of eosinophils into the esophageal epithelium, recent studies have highlighted the potential importance of other cell types including mast cells [5] and basophils [6] suggesting that accumulation of eosinophils into the esophageal epithelium may represent a final common pathway in EoE pathobiology. Interestingly, increased mast cell burden associated with EoE has been shown to respond to treatments including topical steroids [7] and food elimination diets [8]. Furthermore, treatment of pediatric EoE patients with anti-IL5 monoclonal antibody, mepolizumab, led to a reduction in esophageal eosinophilia, as well and significant reduction in mast cells [9].

In addition to their ability to release proteases and prime host Th2-type responses both mast cells and basophils produce and secrete histamine [9–12], a biologic amine with well-characterized roles in allergic responses. Elevated histamine levels have been reported in allergic conditions such as asthma and atopic dermatitis [13]. During allergic inflammation, histamine alters vascular permeability [14] enhances cytokine production by epithelial cells [15], and epidermal keratinocytes [16], promotes migration of inflammatory cells to sites of allergic inflammation [17], and disrupts barrier function [18]. The diverse effects of histamine signaling are mediated by binding to G-protein-coupled receptors of which there are 4 subtypes: HR types 1 (H1R), 2 (H2R), 3 (H3R) and 4 (H4R). While H1R and H2R are expressed by numerous cell types including epithelial cells and fibroblasts, H3R and H4R are primarily expressed in the central nervous system (CNS) and inflammatory cells, respectively [19]. Of the four characterized HRs, H1R, H2R, and to a limited extent H4R, are expressed by epithelial cells of the proximal gut [20].

Antihistamines have been used for several decades for the treatment of allergic disorders, and H1R antagonists are used as first-line therapy for several conditions including allergic rhinitis, allergic conjunctivitis, and urticaria [21]. H1R antagonism results in blockade of histamine induced epithelial cell release of pro-inflammatory cytokines such as GM-CSF [16], IL-6, and IL-8 [22], and H1R mediated disruption of barrier function in human skin [18]. Emerging evidence from numerous animal studies also support the role of H4R receptor as a prominent player in allergic inflammation [13,22–24]. More importantly, studies in humans have identified altered HR expression during allergic rhinitis [25] and irritable bowel syndrome [20]. However, the expression and function of HRs in the esophagus and their possible role in the pathogenesis of EoE remains unknown.

In this study, we characterize the expression of H1R, H2R, H3R and H4R in the human esophagus and show that expression of H1R, H2R and H4R are upregulated in active EoE. Using immortalized and primary human esophageal epithelial cell lines, we investigate the role of histamine signaling in upregulation of physiologically relevant esophageal epithelial cytokine secretion *in vitro* and identify the specific HRs important for this response in the esophagus.

## Materials and Methods

### Human subjects

All research involving human subjects was approved by the Institutional Review Board at The Children’s Hospital of Philadelphia (CHOP Protocol #7737). Following written informed consent obtained from each subject’s parents or legal guardians, 2–4 additional pinch biopsies were obtained from the distal esophagus during routine diagnostic esophagogastroduodenoscopy (EGD). Consistent with recently published clinical guidelines, the diagnosis of EoE was made by the histologic presence of 15 or more esophageal epithelial eosinophils per high powered field (hpf), hyperplasia of the basal epithelium, and the absence of tissue eosinophilia in the distal GI tract [1]. Subjects who met clinical and pathologic criteria for EoE were designated as “active EoE” (≥15 eos/hpf). At CHOP, pediatric EoE patients undergo follow up endoscopy with biopsies at various intervals following treatment interventions (e.g. dietary change, swallowed steroids). Subjects with known EoE whose follow-up biopsies revealed resolution of histologic inflammation and eosinophilia were designated as “inactive EoE” (<15 eos/hpf). Control subjects had no histopathologic abnormalities in the esophagus and distal GI tract, and did not carry a previous diagnosis of EoE. Subjects were excluded from recruitment if they carried a diagnosis of IBD, celiac disease, GI bleeding, or any other acute or chronic intestinal disorders. All subjects in this study were treated with a proton pump inhibitor (PPI) for at least 4 weeks prior to EGD. Patient characteristics and pertinent clinical history are outlined in **Table 1.**

**Table 1:** Characteristics of Pediatric Subjects.

**Phenotype**	**Subject ID#**	**Sex**	**Age**	**Indication for EGD**	**History of atopy**	**Diet or Steroid Treatment**	**Peak Eos/hpf**
**Non-EoE control**	1	M	10y 4m	AP	RAD, AR, AD, FA	DR for FA	0
	2	F	16y 7m	GERD	None	None	0
	3	F	10y 0m	GERD, AP	RAD, AR	NS	0
	4	M	11y 3m	AP	RAD	None	0
	5	M	14y 8m	vomiting	RAD	IS	0
	6	F	13y 9m	AP, nausea	AD	None	0
	7	M	4y 0m	AP, vomiting	RAD, AD	IS	0
	8	M	9y 5m	FTT	RAD	IS, NS	0
	9	M	10y 6m	AP, GERD	AD	NS	0
	10	M	7y 8m	Vomiting	None	None	0
	11	F	15y 2m	Dysphagia	AD	None	0
	12	M	2y 9m	Feeding difficulty	RAD, AD	IS	5
	13	M	1y 1m	Vomiting	None	IS	0
	14	M	2y 1m	Feeding difficulty, FA	AD, FA	DR for FA	2
	15	M	2y 9m	Feeding difficulty	RAD, AD	IS	0
	16	F	13y 1m	AP, vomiting	None	None	0
	17	M	2y 9m	FTT	FA	DR	0
	18	M	2y 9m	Feeding difficulty	RAD	None	0
	19	M	5y 5m	AP, feeding difficulty	None	None	0
	20	M	5y 5m	Feeding difficulty	AR	NS	0
	21	M	10y 8m	AP, diarrhea, vomiting	RAD, AR, AD, FA	IS	0
	22	F	15y 0m	GERD	None	None	0
	23	M	12y 3m	Dysphagia, GERD, vomiting	AD	None	8
**Inactive EoE**	1	M	14y 7m	AP	RAD, FA	DR, IS, SS	0
	2	M	4y 9m	EoE f/u	RAD, AD, RA, FA	DR, IS, SS	0
	3	M	9y 0m	EoE f/u	RAD, AD, AR, FA	DR, IS, NS	2
	4	M	5y 10m	EoE f/u	RAD, AR, FA	DR	0
	5	F	3y 5m	EoE f/u	FA	DR	0
	6	F	16y 10 m	EoE f/u	RAD, AR, FA	SS	0
	7	M	4y 5m	EoE, GERD, food refusal	RAD, AD, AR, FA	DR, IS, NS	0
	8	M	12y 3m	EoE f/u	FA	DR	3
	9	M	11y 7m	EoE f/u, dysphagia	RAD, AD, FA	ED, IS, NS, SS	2
	10	M	5y 8m	EoE f/u	RAD, AD, AR, FA	DR	0
	11	M	10y 1 m	EoE f/u	RAD, AD	SS	6
	12	M	8y 4m	EoE f/u	RAD, AD, FA	DR, IS, NS, SS	0
	13	M	9y 8m	EoE f/u, AP	FA	NS	0
	14	M	7y 7m	EoE f/u	FA	DR, NS	3
	15	M	7y 6m	EoE f/u	RAD, AD, FA	DR, IS	0
	16	M	9y 5m	Dysphagia, AP	RAD, AR, FA	ES, NS, SS	10
	17	M	9y	EoE f/u	RAD, AR, FA	DR, IS	4
	18	M	3y 3m	Vomiting/reflux	RAD, FA	DR, IS	0
	19	M	10y 0m	AP	RAD, AR, FA	IS, NS	1
	20	F	16y 2m	EoE f/u	RAD, AR, FA	DR, IS, NS	0
	21	M	5y 2m	EoE f/u	RAD, AR, FA	DR, IS	2
	22	M	6y 0m	EoE f/u	RAD, FA	DR, IS, NS	0
	23	F	8y 7m	EoE, AP	AR	None	0
	24	M	8y 6m	EoE f/u	AR, FA	DR, SS	10
	25	F	15y 10m	EoE f/u	AR, FA	DR, NS	0
	26	M	15y 2m	EoE f/u	No	SS	3
**Active EoE**	1	F	13y 10m	EoE f/u	AR, AD	DR, IS	100
	2	M	14y 4m	EoE f/u, GERD	RAD, FA	DR, SS	40
	3	M	3y 10m	EoE f/u, emesis	RAD, AR, AD, FA	DR, IS, NS	75
	4	F	16y 4m	EoE f/u	RAD, AR, FA	SS	20
	5	F	5y 8m	EoE f/u	AD, FA	DR	20
	6	M	12y 0m	EoE f/u	FA	DR	28
	7	M	12y 11m	EoE f/u, AP, heartburn, regurgitation	RAD, AR, FA	DR, IS, NS, SS	50
	8	M	8y 10 m	EoE f/u	RAD, AR, AD, FA	DR, IS, NS	75
	9	M	7y 8m	EoE f/u	RAD, AD, FA	DR, IR, SS	25
	10	M	16y 3m	Food impaction, dysphagia	AD	None	20
	11	M	9y 0m	EoE f/u	RAD, AR, FA	DR, SS, IS	50
	12	M	11y 7m	EoE f/u	RAD, FA	IS, NS, SS	75
	13	M	4y 0m	EoE f/u	AR, AD, FA	DR	50
	14	M	5y 1m	EoE f/u	RAD, AR, AD, FA	DR	100
	15	M	16y 9m	EoE f/u, dysphagia	RAD, AR, FA	DR, SS	85
	16	M	4y 9m	EoE f/u, regurgitation	RAD, FA	DR, IS	15
	17	M	12y 4m	EoE f/u	RAD, AD, FA	DR	50
	18	M	2y 11m	EoE f/u	AD	DR	20
	19	F	1y 10 m	EoE f/u, regurgitation, emesis	RAD, AD, FA	ED	25
	20	M	6y 0m	Feeding problems	RAD, AD, FA	DR, IS, NS	50
	21	F	10y 11m	EoE, AP	RAD, AR, FA	DR, NS	50
	22	M	11y 2m	Dysphagia, weight loss	AD	None	36

Characteristics of pediatric subjects used for quantifying HR mRNA expression, including the clinical indication for EGD, history of atopic disease, treatment for atopy, and peak number of eosinophils per high power field at the time of endoscopy.

**AD**: atopic dermatitis

**AP**: abdominal pain RAD: reactive airway disease

**AR**: allergic rhinitis

**DR**: dietary restrictions

**ED**: elemental diet

**EoE f/u**: EoE follow up

**FA**: food allergies (by skin prick or patch testing)

**GERD**: gastroesophageal reflux disease

**IS**: inhaled steroid

**NS**: nasal steroid

**SS**: swallowed steroid

Peak eosinophil numbers, mean +/- sd (median, range) in non-EoE controls (n = 23), inactive EoE (n = 26), and active EoE (n = 23) were 0.65+/-1.94 (0, 0–8), 1.77 +/- 2.9 (0, 0–10), and 48.14 +/- 26.62 (50, 15–100) respectively. Mean age +/- sd (range) of non-EoE, inactive EoE and active EoE cohorts were 8.6 +/- 5.07 (1.1–16.6), 9.12 +/- 4.01 (3.3–16.8), and 9.45 +/- 4.68 (1.8–16.8) respectively. 74% non-EoE, 96% inactive and 100% active subjects had a history of atopy such as allergic rhinitis (AR), atopic dermatitis (AD) and allergic airway disease (RAD). EoE subjects were managed with dietary restrictions (54.2%), swallowed steroid treatment (10.4%), or a combination of both (18.8%). Approximately 43.5% of the non-EoE control subjects were also on steroid treatments such as nasal or inhaled steroids for other atopic condition.

### Primary human esophageal epithelial cell lines

Biopsy samples were placed in ice cold Hank’s buffer, for isolation of esophageal epithelial cells within 30 minutes of harvest. Samples were transferred to dispase (BD Biosciences, 50 u/mL) at 37° C for 20 minutes, followed by trypsinization at 37°C for 20 minutes. Trypsin was inactivated using soybean trypsin inhibitor (Sigma, St. Louis, MO) and biopsy samples were transferred into a cell strainer. Epithelial cells were washed through the strainer with additional PBS. Cell suspensions were centrifuged at 1000 RPM for 5 minutes. Cell pellets were re-suspended in complete KSFM containing fungizone (GIBCO, Grand Island, NY) (1:500), and seeded in tissue culture dishes.

### Cell culture

EPC2-hTERT and primary esophageal epithelial cells (EPC) were grown at 37°C in a humidified 5% CO_2_ incubator and were maintained in keratinocyte serum-free media (KSFM, Life Technologies, Grand Island, NY) supplemented with epidermal growth factor (1ng/mL), bovine pituitary extract (50μg/mL), penicillin (100 units/mL), and streptomycin (100μg/mL) (Invitrogen, Carlsbad, CA).

### Immunofluorescence

Formalin fixed paraffin embedded human esophageal biopsy sections were rehydrated in sodium citrate buffer and blocked with Startingblock T20 blocking buffer (Thermo scientific, Rockford, IL). Sections were then incubated with rabbit anti-H1R (1:100, Santa Cruz, Santa Cruz, CA), goat anti-H2R (1:200, Santa Cruz), or rabbit anti-H4R (1:100, Santa Cruz) diluted in PBT overnight at 4°C. Mouse anti-MBP (1:100, Millipore Temecula, CA) was used to co-stain for eosinophils. After washing with PBS, Cy3 or Cy2-conjugated secondary antibody was applied for 2 hours at room temperature (1:100, Jackson Immunoresearch West Grove, PA). Cells were washed in PBS and stained with 4,6-diamidino-2-phenylindole (1:10,000, Invitrogen) according to manufacturer’s recommendations. Coverslips were applied after the application of mounting media (Biomeda, Foster city, CA), and sections were imaged using Olympus BX51 fluorescent microscope.

### *In vitro* esophageal epithelial stimulation

Esophageal epithelial cells were seeded at a density of 1 X 10^5^ cells/mL in 12 well plates one day prior to stimulation. Confluent cells were cultured in 100uM histamine (Sigma) for 24 hrs and the supernatants were assayed for target protein secretion. For priming experiments, cells were incubated in 10ug/ml poly (I:C) (Invivogen, CA) for 24 hrs prior to stimulation with histamine. For inhibition studies, cells were pre-treated for 1 hr with the H1R antagonist pyrilamine (100uM, Sigma), or H2R antagonists cimetidine (100uM, Sigma), or ranitidine (100uM) (GlaxoSmithKline) prior to histamine stimulation.

### Quantitative RT-PCR

RNA was isolated from human esophageal biopsies using Mirvana miRNA isolation kit (Ambion, Carlsbad, CA). RNA was isolated from EPC2-hTERT and primary esophageal epithelial cells using an RNeasy kit (Qiagen, Valencia, CA) according to manufacturer’s recommendations. RNA samples were reverse transcribed using a high capacity cDNA reverse transcriptase kit according to manufacturer’s instructions (Applied Biosystems, Foster City, CA). Pre-formulated Taqman Gene Expression Assays were purchased from Applied Biosystems for H1R (Hs00185542_m1), H2R (Hs00254569_s1), H3R (Hs00200610_m1), H4R (Hs00222094_m1), and GAPDH (4352934E). Quantitative RT-PCR was performed using the Taqman Fast Universal PCR Master Mix kit (Applied Biosystems) and reactions were performed in triplicate using 96-well optical plates on a StepOnePlus Real-Time PCR System (Applied Biosystems). GAPDH was used as an endogenous control to normalize the samples using the ΔΔC_T_ method of relative quantitation, where C_T_ is the threshold cycle.

### ELISA

Following stimulation, cell supernatants were collected and stored at -80°C until later use. Cytokine levels in supernatants were quantified using ELISA kits purchased from R&D systems (R&D Systems, MN) (GM-CSF, IL-8, TNFα, and eotaxin-3) and eBiosciences (San Diego, CA) (TSLP). Assays were performed according to manufacturer recommendations.

### Statistical analysis

Kruskal Wallis analysis with Dunn’s post test and Mann Whitney U test were used to analyze mRNA data from biopsy samples. Two tailed student t-test and One way ANOVA with Bonferroni comparison was used for in-vitro studies. All analyses were performed in GraphPad Prism 5.

## Results

### Histamine receptors are expressed in the human esophagus *in vivo*

**Fig 1:**
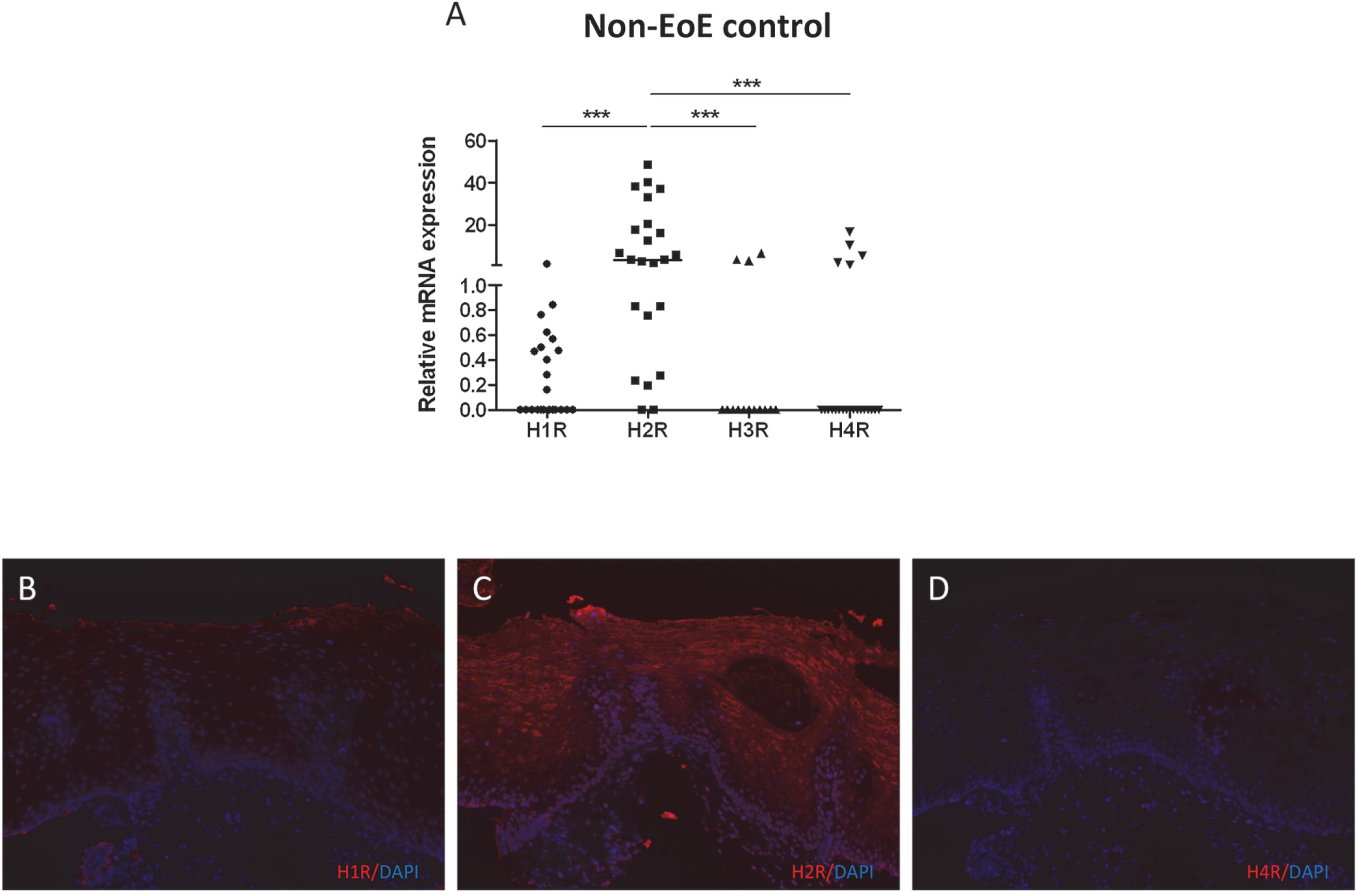
H1R and H2R are expressed in human esophageal mucosal biopsies. Quantification of HR expression in esophageal biopsies from nonEoE control biopsies (**A**). Kruskal wallis test and Dunn’s multiple test was used to determine significance between groups. Horizontal lines represent medians of each data set (***p<0.001). Immunofluorescence for H1R (**B**), H2R (**C**), and H4R (**D**) in esophagus of non-EoE control, subjects (original magnification 200x).

Although HR expression has been studied in epithelial cells in other models such as the bronchial epithelium [15] and epidermal keratinocytes [16,26], HR expression in the esophageal epithelium remains unknown. We first sought to characterize the constitutive HR expression in the esophagus by quantifying H1R, H2R, H3R, and H4R mRNA in histologically normal esophageal biopsies from subjects who did not carry a diagnosis of EoE (N = 23). Patient characteristics are outlined in Table 1. H1R was detected in 11/23 (47.8%) and H2R was detected in 21/23 (91.3%) biopsies (**Fig. 1A**). Both H3R and H4R mRNA expression was minimal, or not detected in the majority of esophageal biopsies. Overall, H2R was the most abundantly expressed receptor subtype in the esophageal biopsies (p<0.001 vs. H1R, H3R, and H4R) (**Fig. 1A**).

We localized esophageal H1R, H2R, and H4R expression with immunofluorescence. H1R was faintly distributed throughout the esophageal epithelium (**Fig. 1B**). Consistent with its mRNA expression in esophageal biopsies, H2R was the most abundantly expressed HR throughout the esophageal epithelium (**Fig. 1C**). Immunofluorescent detection of H4R staining was faint, and was notably clustered within the epithelial papilla (**Fig. 1D**).

### Expression of H1R, H2R, and H4R is upregulated in esophageal biopsies of actively inflamed EoE subjects

HR expression is altered in the epithelium during allergic conditions [25] suggesting that this may be a hallmark of allergic diseases including EoE. We next determined whether HR expression was altered during EoE associated inflammation by comparing HR mRNA levels in non-EoE controls (N = 23), inactive EoE (N = 26), and active EoE subjects (N = 22). Patient cohorts were stratified based on the current guidelines for EoE diagnosis [1] (**Table 1**).

**Fig 2:**
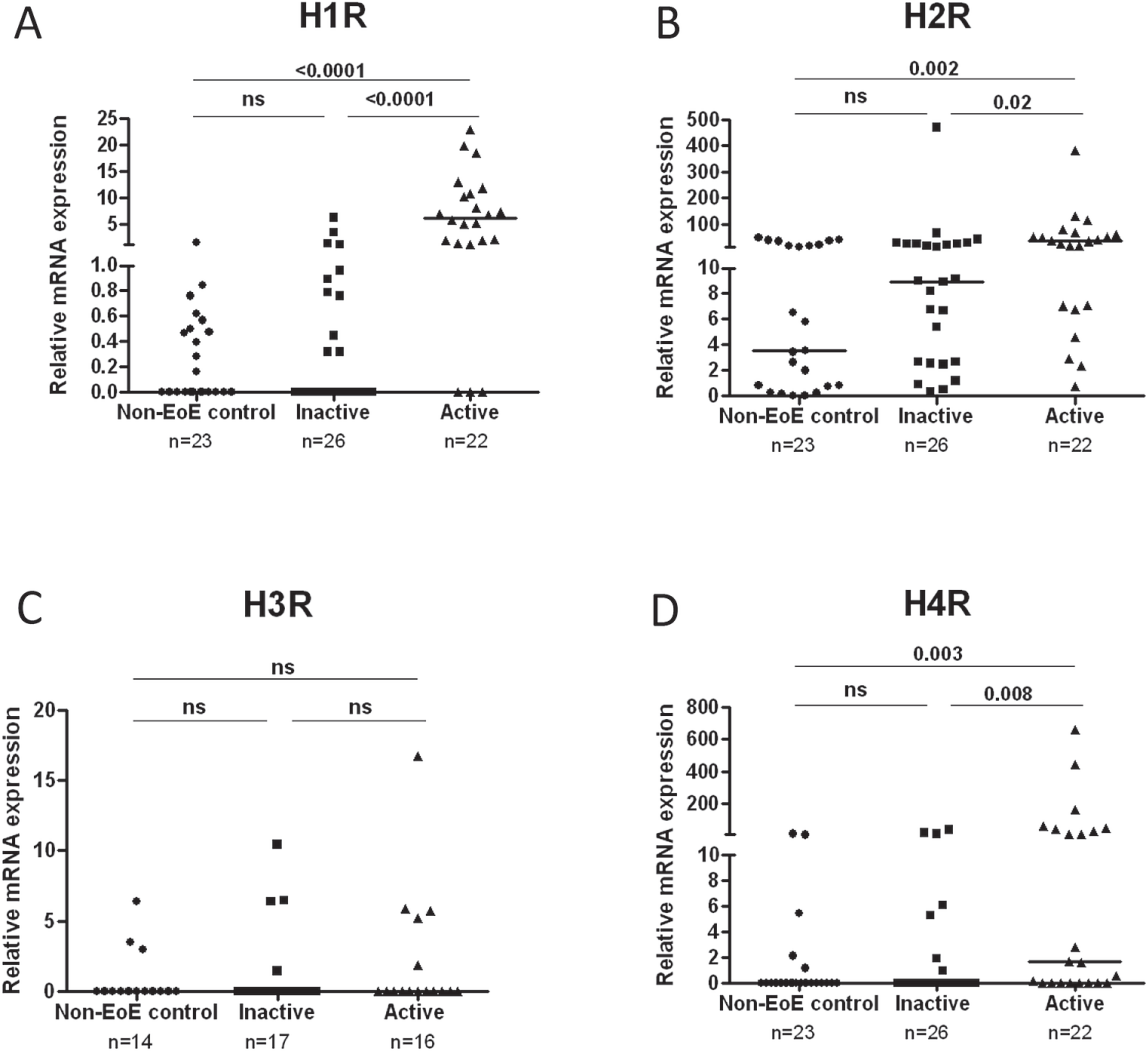
Expression of H1R, H2R, and H4R 4 are increased in esophageal biopsies in actively inflamed EoE. Comparison of H1,H2, H3, and H4 receptor expression in non-EoE control, inactive EoE and active EoE biopsies, using Mann-Whitney test (**A-D**). Horizontal bars represent median of each data set. Horizontal lines represent median of the data set.

Interestingly, H1R, H2R, and H4R expression levels were significantly elevated in active EoE subjects compared to non-EoE control and inactive EoE subjects (**Fig. 2A, B, and D**). Additionally, expression of all HRs in inactive EoE biopsies was similar to that in non-EoE control biopsies. H3R expression was unchanged in active EoE subjects compared to either non-EoE control or inactive EoE subjects (**Fig. 2C**).

### Infiltrating inflammatory cells express HRs, and tissue eosinophil counts correlate with mucosal HR expression during active inflammation in EoE

**Fig 3:**
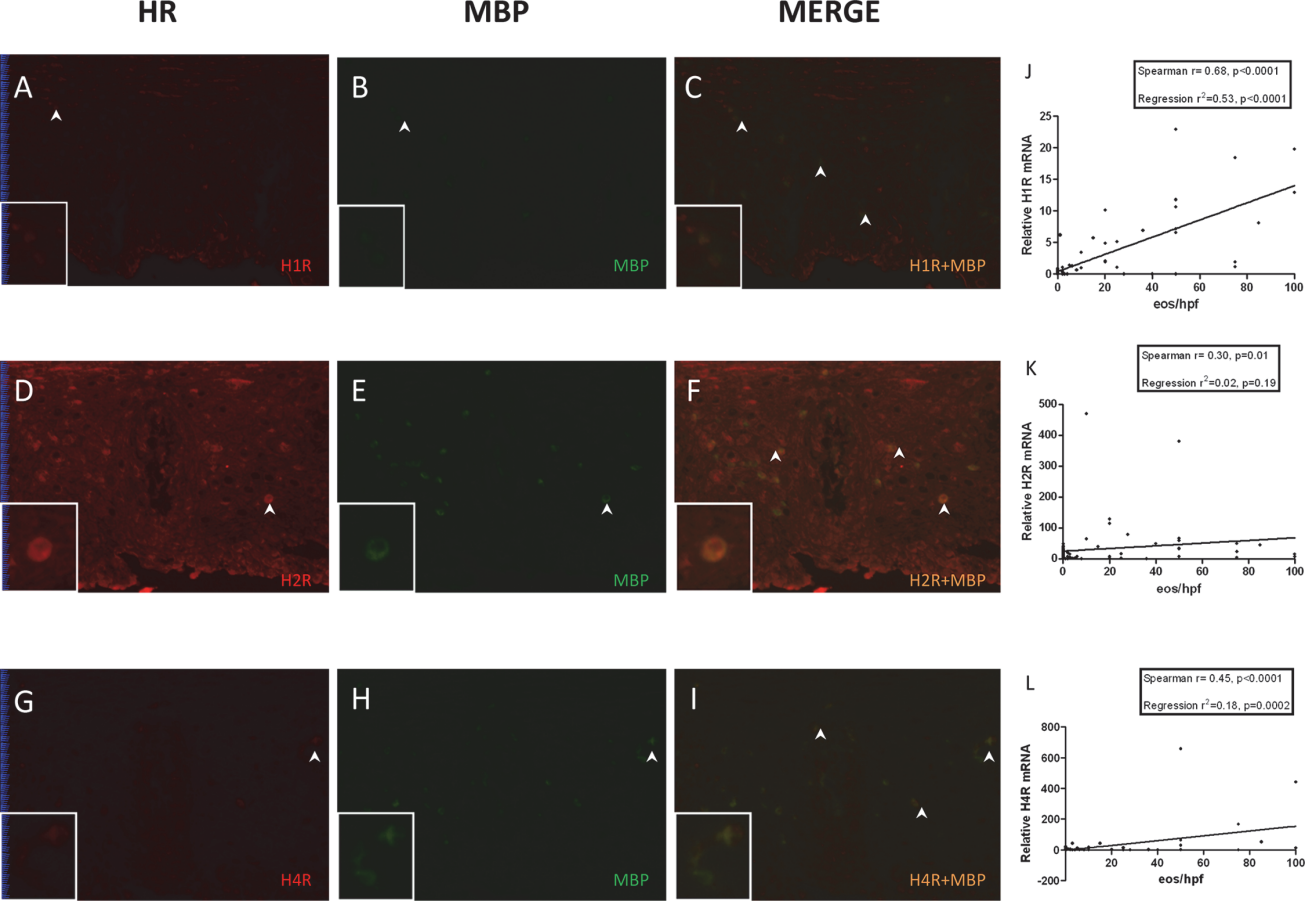
Infiltrating eosinophils express HR and correlate with mucosal HR expression in active EoE. Immunofluorescent localization of MBP and H1R (**A-C**), H2R (**D-F**) and H4R (**G-I**) in esophageal biopsies from active EoE subject (original magnification 200x). Arrows indicate MBP-HR colocalization. Representative cells with MBP-HR colocalization are magnified in inserts. Correlation of H1R (**J**), H2R (**K**), and H4R (**L**) mRNA in human esophageal biopsies to peak tissue eosinophil count per hpf, using Spearman correlation and linear regression analyses.

HRs are also expressed by various inflammatory cells including eosinophils, dendritic cells, and T cells [27,28]. To determine the contribution of eosinophils to the altered HR expression observed in active EoE subjects, we performed dual staining of esophageal biopsies for HRs and major basic protein (MBP), a granular protein expressed by eosinophils and basophils [29]. MBP expression was absent in inactive EoE and non-EoE control biopsies (data not shown) but was detected in active EoE biopsies (**Fig. 3B, E, H**). Subsets of MBP positive cells also expressed H1R, H2R and H4R as evident from their co-localization (**Fig. 3 C, F, I**). Based upon these results, we next determined whether tissue eosinophilia in EoE correlated with HR expression in esophageal biopsies. Shown in **Fig. 3J-L**, peak eosinophil numbers per hpf correlated positively with H1R, H2R, and H4R expression. H1R had the strongest correlation to eosinophils numbers among the three receptor subtypes, followed by H4R and H2R. These findings suggest that a significant portion of upregulated histamine receptor expression detected in active EoE biopsies is derived from infiltrating inflammatory cells.

### HRs are expressed by human esophageal epithelial cells *in vitro*

**Fig 4:**
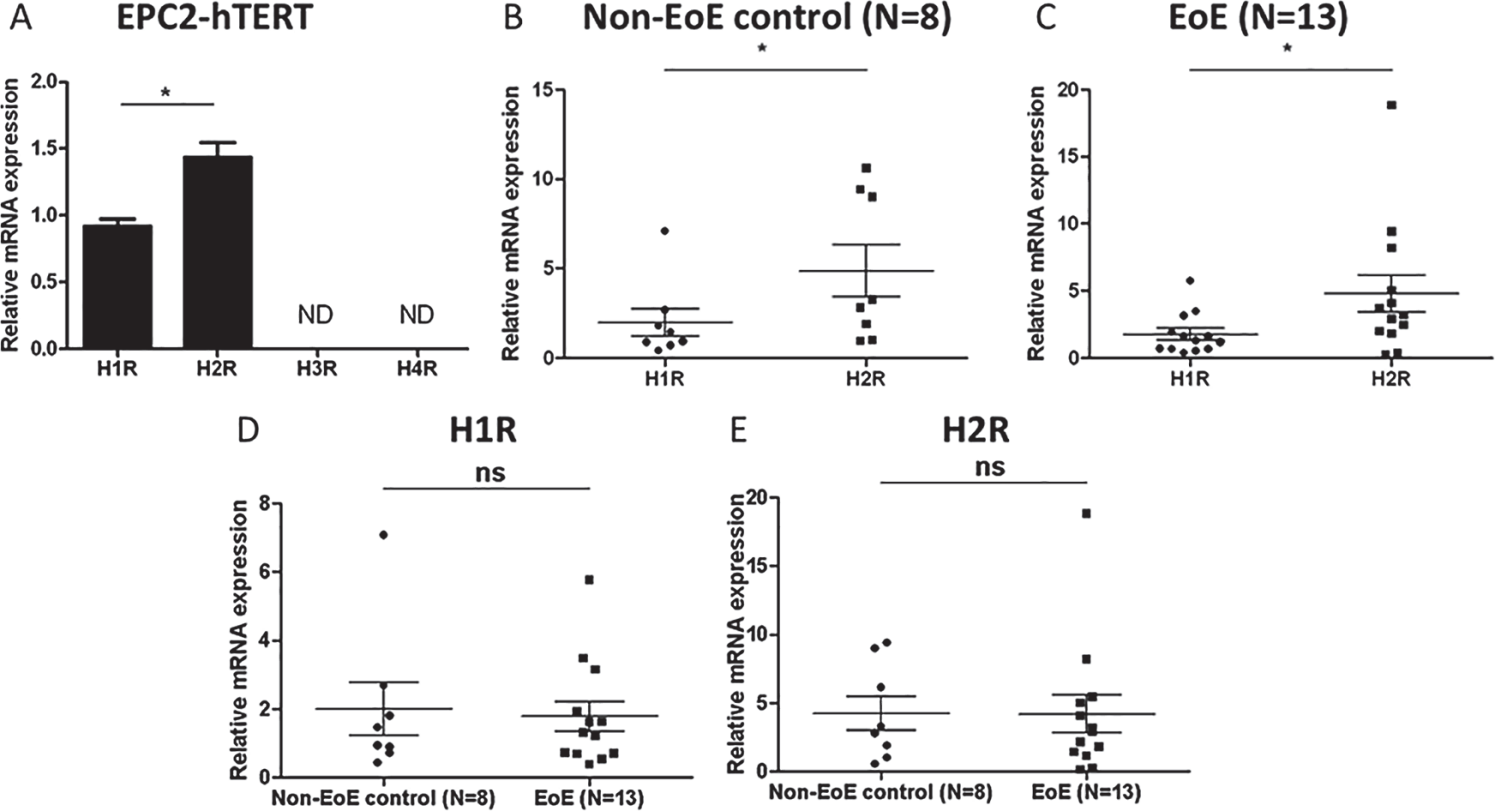
Primary and immortalized esophageal epithelial cells express H1R and H2R. HR mRNA expression in human esophageal epithelial cell line, EPC2-hTERT (**A**) and primary esophageal epithelial cell lines derived from non-EoE control (**B,D,E**) and EoE (**C,D,E**) subjects. **A**: Data represent mean +/- SEM for n = 3. **B-E**: horizontal bars represent median with IQR. Mann-Whitney test was employed to quantify significance. *p<0.05, ns = not significant.

Recognizing that the quantification of HR expression in mucosal biopsies may reflect HR expression by a number of cell types (epithelial, inflammatory, and stromal), we next sought to elucidate the specific role of esophageal epithelial cells in histamine signaling in EoE. Constitutive expression of HRs in the immortalized human esophageal epithelial cell line, EPC2-hTERT and in primary esophageal epithelial cell lines derived from EoE and non-EoE esophageal biopsies was quantified by quantitative RT-PCR. While both H1 and H2 receptors were constitutively expressed by EPC2-hTERT (**Fig. 4A**), non-EoE control (n = 8) (**Fig. 4B**), and EoE (n = 13) (**Fig. 4C**) cell lines, neither H3R nor H4R were detected in any of the studied cells (data not shown). Consistent with histamine expression patterns within esophageal biopsy samples, expression of H2R was significantly greater than H1R in all cell lines (**Fig. 4A-C**). Importantly, relative expression of H1R and H2R was not significantly different in EoE compared to non-EoE control cell lines. (**Fig. 4D, E**). Further stratification of EoE cells into inactive EoE (N = 4) and active EoE (N = 9) did not show a significant difference in receptor expression either (data not shown). Based on these similarities in HR expression between primary and EPC2-hTERT esophageal epithelial cells, subsequent experiments were performed in EPC2-hTERT and one representative cell line from each of the EoE and non-EoE cohorts.

### Histamine induces GM-CSF, IL-8 and TNFα secretion by esophageal epithelial cells *in vitro*

We next sought to understand the physiological relevance of HR expression in the esophageal epithelium in EoE. Previous studies have reported overexpression of numerous pro-inflammatory cytokines in the esophageal epithelium during EoE, including IL-5, IL-13, eotaxin-3, TSLP, IL-8, TNFα, and IL-6 [30–32]. Histamine induces pro-inflammatory cytokines including GM-CSF, IL-8, and IL-6 [22] in epithelial cells and promotes eosinophil chemotaxis and adhesion *in vitro* [28]. Furthermore, H1 inhibition has demonstrated suppression of histamine induced pro-inflammatory cytokines such as GM-CSF [16], IL-6 and IL-8 [22]. We therefore hypothesized that histamine stimulation of esophageal epithelial cells *in vitro* would induce the secretion of pro-inflammatory cytokines relevant to EoE pathogenesis.

**Fig 5:**
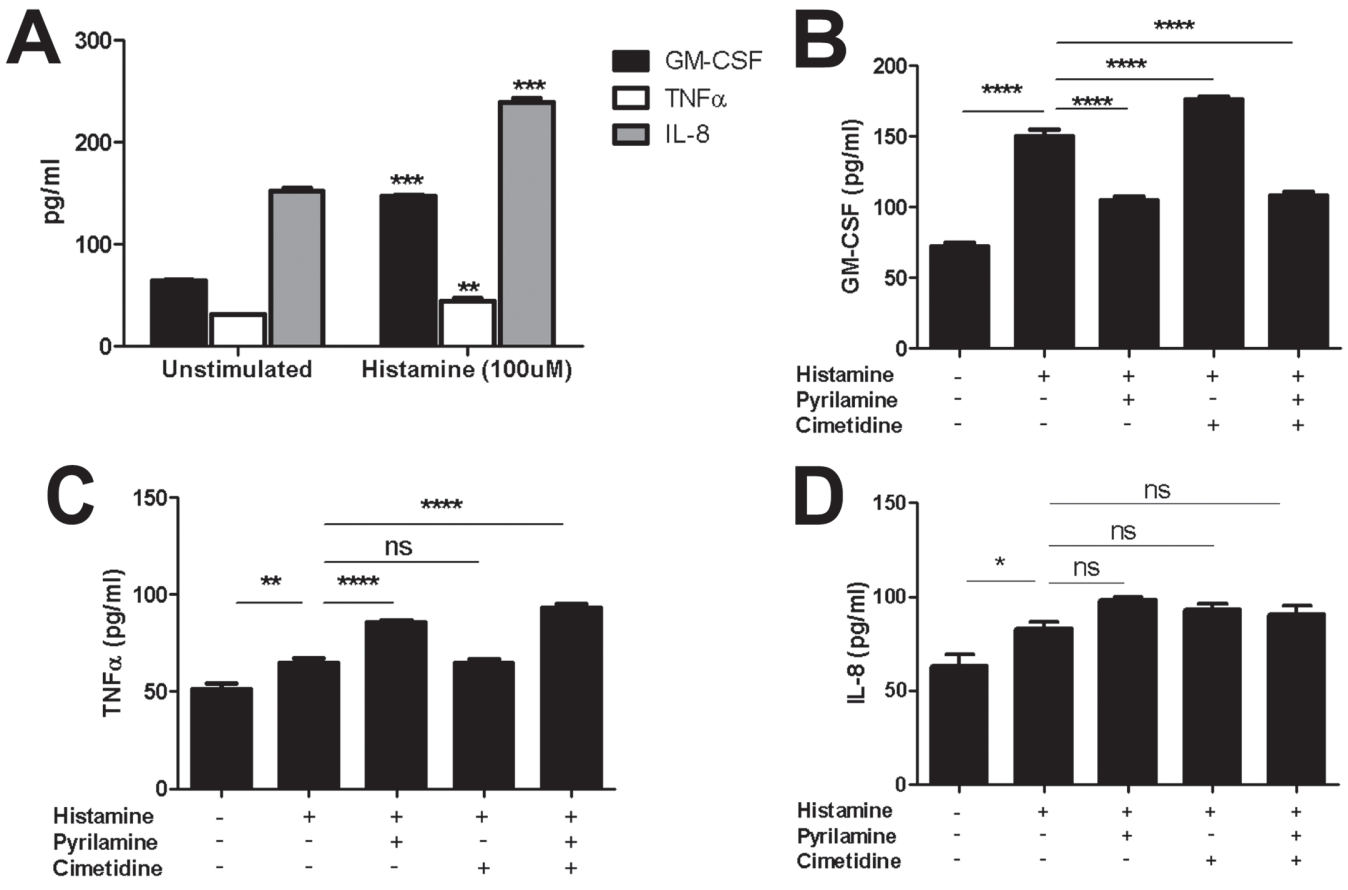
Esophageal epithelial cells produce cytokines in response to histamine stimulation *in vitro*. Quantification of GM-CSF, TNFα, and IL-8 (pg/mL) secretion by histamine stimulated EPC2-hTERT cells (100uM histamine for 24 hrs). Two-tailed student t-test was used for statistical analysis. **p<0.005,***<0.0005. Effect of pretreatment with H1R antagonist pyrilamine or H2R antagonists (cimetidine and ranitidine) prior to histamine stimulation upon cytokine secretion in EPC2-hTERT cells (***B-D***). One way ANOVA with Bonferroni post-test was used for statistical analysis. *p<0.05, **p<.01,****p<0.0001. ns = not significant. Data are represented as the mean +/- SEM of n = 6.

Histamine stimulation induced robust secretion of GM-CSF and IL-8 and a mild induction in TNFα secretion by EPC2-hTERT cells (**Fig. 5A**), and primary EoE and non-EoE cells (data not shown). Surprisingly, histamine stimulation of EPC2-hTERT and primary cells did not induce either thymic stromal lymphopoetin (TSLP) or eotaxin-3 (CCL26) secretion, which are epithelial derived cytokines prominently linked to EoE pathogenesis [6,30,33] (data not shown).

In order to determine the specific HRs involved in inducible cytokine expression in esophageal epithelial cells, we pre-treated EPC2-hTERT cells with pharmacological inhibitors of H1R and H2R prior to histamine stimulation. H3 and H4 receptor inhibitors were not studied based upon the absence of these receptors in primary or immortalized esophageal epithelial cells. Pretreatment with H1 inhibitor pyrilamine suppressed histamine-mediated GM-CSF expression, whereas H2 receptor antagonists cimetidine and ranitidine had a very mild inducible effect (**Fig. 5B**). TNFα expression was mildly induced by H1R inhibition while H2R inhibition did not have any effect (**Fig. 5C**). Interestingly, neither H1R nor H2R inhibition suppressed histamine mediated IL-8 expression in EPC2-hTERT cells (**Fig. 5D**).

### Toll-like receptor 3 stimulation enhances epithelial responsiveness to histamine *in vitro*

Based on the increased HR expression observed in active EoE biopsies, and histamine mediated cytokine expression in epithelial cells *in vitro*, we hypothesized that epithelial sensitivity to histamine might be enhanced in a setting of inflammation and subsequent increased HR expression. H1R expression and function can be induced by innate immune signaling through toll-like receptor 4 (TLR4) in human coronary artery endothelial cells [34]. We have previously reported that TLR3 is the most highly expressed and functional TLR in human esophageal epithelial cells [35], and others have shown that TLR3 ligand stimulation induces expression of epithelial cytokines relevant to EoE [36]. We therefore hypothesized that stimulation of epithelial cells with the TLR3 agonist, polyinosinic/polycytidylic acid (polyI:C) would induce epithelial expression of HRs and subsequently enhance esophageal epithelial sensitivity to histamine *in vitro*.

**Fig 6:**
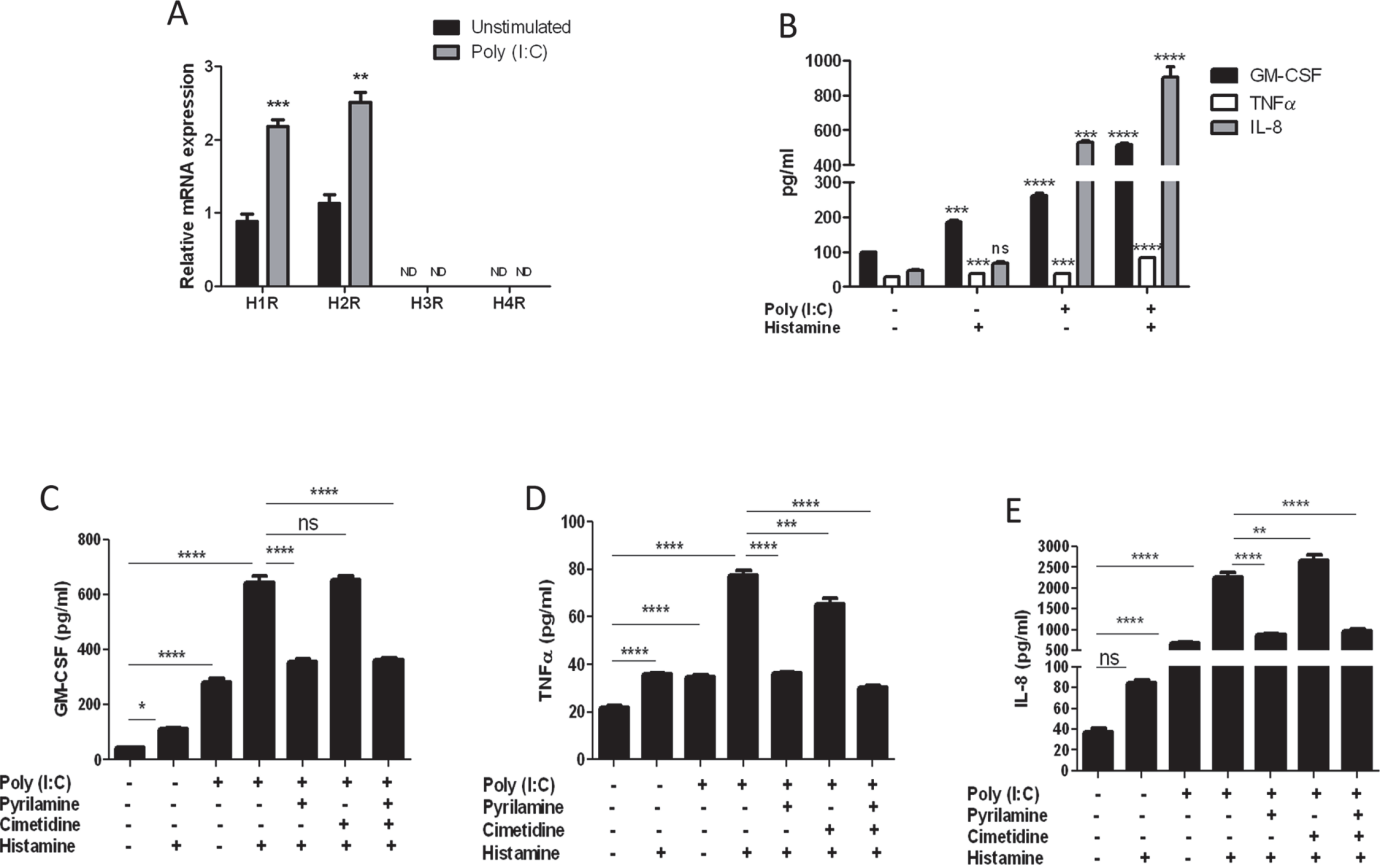
Toll-like receptor agonist poly (I:C) induces expression of H1R and H2R and enhances epithelial histamine responsiveness in EPC2-hTERT cells. (**A**) Expression of H1R, H2R, H3R, and H4R following stimulation with poly (I:C) (10ug/ml) for 24 hrs. A Student t-test was used to calculate significance **p<0.005,***p<0.0005, ND = not detected. (**B**) Effect of poly(I:C) priming upon histamine-induced cytokine expression in EPC2-hTERT cells. One way ANOVA with Bonferroni post test was used for statistical analysis. ***p<0.001, ****p<0.0001. ns = not significant. Data represented as mean +/- SEM of n = 3. (**C-E**) Poly (I:C)—histamine induced cytokine secretion in EPC2-hTERT cells with/without H1R, H2R antagonism. One way ANOVA with Bonferroni post test was used for statistical analysis. *p<0.05, **p<0.01, ***p<0.001****p<0.0001. ns = not significant. Data represented as mean +/- SEM of n = 6.

Stimulation of EPC2-hTERT cells with poly (I:C) led to robust induction in H1R and H2R mRNA expression (**Fig. 6A**). Neither H3R nor H4R mRNA was induced by poly (I:C). To determine whether poly (I:C) mediated induction in HR expression resulted in enhanced sensitivity to histamine stimulation, esophageal epithelial cells were primed with poly (I:C) for 24 hrs prior to stimulation with histamine, followed by quantification of GM-CSF, TNFα, IL-8, eotaxin 3, and TSLP secretion. Poly (I:C) stimulation alone induced robust secretion of GM-CSF, TNFα, and IL-8, which was synergistically enhanced by subsequent histamine stimulation (**Fig. 6B**). Again, neither eotaxin-3 nor TSLP was induced by combined poly (I:C) and histamine stimulation (data not shown). We next determined the roles of specific HRs in poly (I:C)/histamine mediated cytokine induction using pharmacological inhibitors of H1R and H2R. H3R and H4R inhibitors were not used based upon lack of poly (I:C) inducible expression of these receptors in esophageal epithelial cells *in vitro*. The secretion of GM-CSF, TNFα, and IL-8 in poly (I:C) primed epithelial cells was significantly suppressed by H1R antagonist pyrilamine (**Fig. 6C-E**). The H2R antagonist cimetidine suppressed poly (I:C)-histamine induced TNFα secretion (**Fig. 6D**), though to a lesser degree than pyrilamine alone. The effects of combined pyrilamine with either cimetidine or ranitidine were not significantly different from that of pyrilamine alone, indicating the effect is primarily mediated via H1R.

## Discussion

In the current study, we now show for the first time that H1R, H2R, and H4R, but not H3R, are expressed in human esophageal epithelial biopsies, and their expression is induced in the setting of active inflammation in EoE. Importantly, we demonstrate that H1R and H2R, but not H3R or H4R, are constitutively and inducibly expressed by human esophageal epithelial cell lines *in vitro*, suggesting that inducible esophageal H4R expression *in vivo* in the setting of active EoE may be of non-epithelial origin. Using HR antagonists, our data suggests that histamine-mediated epithelial cytokine secretion, especially that of GM-CSF, is mediated primarily by H1R signaling, despite highest levels of H2R in both esophageal biopsies and esophageal epithelial cell lines. Finally, we demonstrate that priming of esophageal epithelial cells with the TLR3 ligand poly(I:C) enhances epithelial sensitivity to histamine stimulation through primarily H1R and to a lesser degree H2R-mediated mechanisms.

Mucosal expression of H1R, H2R, and H4R was significantly increased in biopsies with active inflammation, compared to both non-EoE control and inactive EoE biopsies. This is consistent with previous reports describing similar alterations in HR patterns in allergic diseases including allergic rhinitis [25] and intestinal motility disorders including irritable bowel syndrome [20]. In contrast to our findings in mucosal biopsies, comparisons of epithelial-specific HR expression between non-EoE control and EoE cohorts *in vitro* were not statistically different and esophageal epithelial cells from EoE and non-EoE subjects had similar cytokine secretion patterns upon histamine stimulation. Together with our observation that HRs are expressed by infiltrating eosinophils in active EoE samples, these observations suggest that increased esophageal epithelial histamine receptor expression in active EoE biopsies may reflect the inflammatory milieu and may not represent an intrinsic defect in HR expression or histamine signaling in epithelial cells.

The precise factors involved in inducing esophageal epithelial histamine receptor expression remain unknown. Mirzahosseini et al. have recently shown that IgE stimulation results in upregulation of H2R in murine bone marrow derived mast cells [37]. However, emerging evidence suggests that EoE is associated with IgG4, and not mediated by IgE[38]. Interestingly, patients with allergic rhinitis have increased H2R expressing regulatory T cells at the peak of pollen season [39]which points towards the possible role of food allergens in promoting HR expression during EoE. It is also not known if HR expression is regulated by Th2 cytokines such as IL5 and IL13 which are overexpressed during active inflammation in EoE.

H2R was the most highly expressed HR in biopsies and epithelial cells *in vitro*. Paradoxically, competitive inhibition of H2R signaling did not significantly suppress histamine-mediated GM-CSF expression. Alonso et al showed a negative cross-regulation mechanism between H1R and H2R in human and CHO cells [40]. This may suggest that higher epithelial H2R expression may be required to compensate for the pathological inflammatory effects induced by H1R signaling. Alternatively, others have shown that histamine signaling via H2R in Peyers patches is protective in murine models of *Yersinia enterocolitica* infection [41] and histamine suppresses LPS-mediated TNFα secretion by human PBMCs [42]. It is therefore plausible that the observed high levels of H2R expression may play a protective role in host innate immunity against pathogens in the esophagus.

H3R is expressed by myocytes and in the central and peripheral nervous system [19]. In the GI tract, H3R agonists promote proliferation of murine gastric and intestinal epithelial cells [43]; however, H3R has not been detected in the human gastrointestinal epithelium [20]. Though we noted a paucity of H3R expression in both esophageal biopsy samples and primary esophageal epithelial cells, it is possible that these results are secondary to sampling bias, as our biopsies likely excluded sampling of esophageal smooth muscle and enteric neurons. In models of human allergic rhinitis, H3R and H1R antagonism reduced histamine- mediated nasal inflammation, which was attributed to antagonists’ action on sympathetic neurons rather than epithelial cells [44]. As longstanding EoE results in esophageal dysmotility, further studies will be critical to fully elucidate the potential role of H3R signaling in EoE.

Although there was no detectable H4R expression in esophageal epithelial cell lines, H4R expression was detectable in biopsies from EoE subjects with active disease. We noted co-localization of H4R and MBP, suggesting that infiltrating eosinophils and basophils may partially account for the detectable H4R signal. Other inflammatory cells including mast cells and T cells [45] may contribute to detectable H4R expression in actively inflamed biopsies. Indeed, H4R is emerging as a potential treatment target in murine models of inflammatory bowel disease (IBD)[45,46], atopic dermatitis [13,47], and allergic airway inflammation [48]. In a guinea pig model of EoE, Yu et al. showed that antigen sensitization and stimulation resulted in H4R dependent mast cell and eosinophil infiltration in the esophagus [49]. Thus, while histamine may not signal through H4R in esophageal epithelial cells *in vitro*, upregulation of H4R in the esophageal epithelium *in vivo* suggests a potential role for H4R signaling in EoE. Further studies are required to understand the role of H4 receptor signaling in EoE pathobiology, particularly since current antihistamine therapies (H1 and H2) are already utilized in approximately 10% of patients diagnosed with EoE patients [1] and do not appear to ameliorate the eosinophilic inflammation in EoE.

Recognizing that the biological triggers of increased epithelial HR expression *in vivo* are incompletely understood, we recapitulated elevated HR expression *in vitro* using the TLR3 ligand and viral mimetic, poly(I:C), which strongly induced H1R and H2R expression. Viral infection is linked to other diseases such as allergic asthma [50] and perennial allergic rhinitis [51]. This is thought to occur through interactive inflammatory mechanisms such as barrier dysfunction, cytokine signaling and immune cell dysregulation ultimately resulting in inflammatory pathway activation including mast cell recruitment and degranulation. While we cannot exclude the involvement of other pattern recognition receptors in esophageal histamine signaling, we and others have previously shown that poly (I:C) stimulation of esophageal epithelial cells *in vitro* leads to the expression of various cytokines relevant to esophageal inflammation including TSLP[36] RANTES [52], and IL-8 [35,53]. Though HRs can be induced in other models by activation of additional TLR signaling pathways including TLR4, we have previously demonstrated that esophageal epithelial cells lack TLR4 expression and that TLR3 is the most abundantly expressed and functional of TLRs in human esophageal epithelial cells [35].

Histamine stimulation induced esophageal epithelial secretion of GM-CSF, IL-8, and TNFα *in vitro*. GM-CSF, though not previously quantified in EoE, plays a critical role in promoting eosinophil migration, survival, activation, and degranulation [54–56] and is a direct downstream target of histamine signaling in many allergic diseases [57,58]. Both IL-8 and TNFα induction have been documented in the esophageal epithelium during active inflammation [31,32]. In models of environmental allergen-induced nasal allergy, histamine induces the expression of IL-8 [59], which can promote eosinophil and basophil chemotaxis [60,61]. Though others have demonstrated that histamine signaling suppresses LPS-mediated TNFα secretion through H2R[42], to our knowledge the direct effect of histamine upon TNFα expression has not been previously reported. TNFα is a cytokine with diverse functions and could be involved in epithelial to mesenchymal transition (EMT) relevant to fibrosis in EoE [62]. Interestingly, in the setting of poly(I:C)-mediated HR upregulation, all three cytokines were significantly enhanced, and were primarily regulated by H1R signaling. Importantly, histamine stimulation, even in the setting of enhanced epithelial sensitivity had no effect upon the epithelial expression of the two key EoE cytokines, TSLP and eotaxin-3. These findings suggest that histamine signaling can significantly contribute to the local inflammatory microenvironment during active inflammation, but is not crucial for the onset of inflammation itself in EoE. Histamine mediated cytokine expression was differentially regulated by histamine receptors depending on priming by poly (I:C). While H1R mediated regulation of GM-CSF was independent of poly (I:C) priming, it was essential for H1R mediated expression of TNFα and IL8. Similarly, poly (I:C) priming was required for H2R mediated TNFα expression. Although poly (I:C) mediated induction of H1R and H2R may account for this observation, the contributions of other pro-inflammatory factors induced by poly (I:C) sensitization cannot be ruled out. Additionally, It has been shown that TLR activation may have a direct effect on G-protein coupled receptor (GPCR) regulating kinases resulting in a TLR-GPCR crosstalk [63]. This crosstalk can play a significant role in our model since histamine receptors are well known GPCRs. Therefore, further studies are required to understand the mechanism underlying histamine signaling in a EoE specific inflammatory microenvironment.

In summary, we show that H1R, H2R, and H4R expression is altered in the esophageal epithelium during active inflammation in EoE, possibly enhanced by the inflammatory microenvironment including infiltrating immune cells. Despite the lack of clinical data supporting the use of H1R antagonists in EoE, our findings suggest that H1R signaling may contribute to local epithelial cytokine responses in the esophagus, highlighting the complexity of the host immune response in EoE. Although H4R was neither constitutively or inducibly expressed by human esophageal epithelial cells, its upregulation in mucosal biopsies warrant future functional studies using animal models to further define the role of H4R in EoE associated inflammation.
